# Ensemble Fractional Sensitivity: A Quantitative Approach to Neuron Selection for Decoding Motor Tasks

**DOI:** 10.1155/2010/648202

**Published:** 2010-02-14

**Authors:** Girish Singhal, Vikram Aggarwal, Soumyadipta Acharya, Jose Aguayo, Jiping He, Nitish Thakor

**Affiliations:** ^1^Department of Biomedical Engineering, Johns Hopkins University, Baltimore, MD 21205, USA; ^2^The Harrington Department of Bioengineering and the Center for Neural Interface Design, Arizona State University, Tempe, AZ 85287-9709, USA

## Abstract

A robust method to help identify the population of neurons used for decoding motor tasks is developed. We use sensitivity analysis to develop a new metric for quantifying the relative contribution of a neuron towards the decoded output, called “*fractional sensitivity*.” Previous model-based approaches for neuron ranking have been shown to largely depend on the collection of training data. We suggest the use of an *ensemble* of models that are trained on random subsets of trials to rank neurons. For this work, we tested a decoding algorithm on neuronal data recorded from two male rhesus monkeys while they performed a reach to grasp a bar at three orientations (45°, 90°, or 135°). An ensemble approach led to a statistically significant increase of 5% in decoding accuracy and 25% increase in identification accuracy of simulated noisy neurons, when compared to a single model. Furthermore, ranking neurons based on the ensemble fractional sensitivities resulted in decoding accuracies 10%–20% greater than when randomly selecting neurons or ranking based on firing rates alone. By systematically reducing the size of the input space, we determine the optimal number of neurons needed for decoding the motor output. This selection approach has practical benefits for other BMI applications where limited number of electrodes and training datasets are available, but high decoding accuracies are desirable.

## 1. Introduction

A Brain-Machine Interface (BMI) uses activities recorded from various motor areas, such as the primary motor, premotor and posterior parietal cortex, to translate neural activities recorded from the brain into commands to control an external device. Traditionally, BMI researchers have used extracellular action potentials from localized cortical sites, primarily in the motor cortex, to provide closed-loop control of a computer cursor [1] or a robotic arm in 3D space [2–4]. More recently, researchers have now begun to use implanted microelectrode arrays, which can simultaneously sample neuronal ensembles from various cortical sites [5–7].

These electrode arrays are surgically placed in cortical regions which are correlated to the motor function. The relevant cortical regions are identified using anatomical guidance, preliminary probing of neural activity and imaging techniques such as FMRI. However, in multichannel recordings only 30%–40% of single units are typically relevant to the motor task [8]; the remaining neurons are either noisy or not task-related. This adds uncorrelated dimensions to the input space, thereby degrading the predictive performance of the decoding filter due to overfitting [9, 10]. Hence, there is a need to develop a metric for evaluating the contribution of neurons selected for BMI tasks. Such a metric would then be used to rank neurons based on relative importance to the task. Selecting a subpopulation of rank-ordered neurons will help prune the input space to a smaller population free of irrelevant neurons. Furthermore, reducing the input space in a multichannel system is also strongly motivated by hardware limitations and increased computational burden in relating the output kinematic variable to the input space.

A neuron selection method can either be looking at a univariate or a multivariate input space. In a univariate approach, each neuron is individually assessed by observing the change in its firing rate with respect to the “no stimulus” period. This method has its origin in classical single electrode neurophysiology experiments wherein a neuron that is found to be unresponsive to the motor task is disregarded. From the perspective of building BMIs, a neuron which exhibits variability of response for different tasks should be retained. This is traditionally done by visual inspection, or by using statistical methods such as ANOVA [11] or information theoretic approaches such as Shannon"s entropy that are not directly related to decoding.

Comparatively, multivariate approaches to neuron selection assess the contribution of a neuron in the presence of the entire neural population. Reducing the entire input space to a subset of task-related features is a classical machine learning problem, and commonly referred to as dimensionality reduction. “Projective” methods such as Linear Discriminant Analysis (LDA) find a linear transformation which maps the original input space to a smaller dimension, while maximizing the separation of different class clusters [12]. These “projective” techniques, however, do not provide any information about individual neurons. Furthermore, the transformed space is a linear combination of the inputs and hence does not reduce the actual number of input signals recorded. “Feature selection” methods, on the other hand, find a subset of original features that are most relevant to the task. Therefore, these methods preserve the meaning of the features while simultaneously reducing the input space. 

Feature selection for BMIs has become an active area of research, with various approaches that attempt to extract the relevant inputs at the same time as training the decoding filter. Several decoding filter models have been used to translate neural activity into the corresponding kinematic variable. These include linear filters such as Wiener filters [1, 4, 6], recursive Bayesian models such as Kalman filters [13–15], and nonlinear filters such as Artifical Neural Networks [4, 16–18]. Training these decoding filters implicitly assigns weights to neurons depending on their relative contribution and importance to mapping the output variable. As has been shown previously [10, 19], one can then interpret the weights and biases of trained input-output models in order to ascertain the importance of neuron in decoding commands for a motor task. Another approach ranks neurons on the basis of an overall decrease in the decoding accuracy as neurons are systematically dropped [7]. A model-based sensitivity analysis however suffers from well-known problem of over fitting (more commonly referred to as model generalization). It is indeed interesting to note the effects of this problem on the ranking of neurons and eventually the performance of a BMI. In [10], Sanchez et al. suggested a new metric to quantify importance of neuron towards decoding using a model-based sensitivity analysis approach. The reference notes that different ranking methodologies gave different results and thus alternative ranking methodologies must be looked in that combined regularization theory to find a smaller subset of neurons that further improves decoding accuracy. In this work we have adapted their approach and suggested improvements to counter the limitations of using a model-based approach by using an ensemble of models. We have demonstrated the benefits of using an ensemble of models over a single model by showing higher decoding accuracy (Figure 6) and better success rate in identifying noisy neurons (Tables 2(a) and 2(b)). 

**Figure 1:**
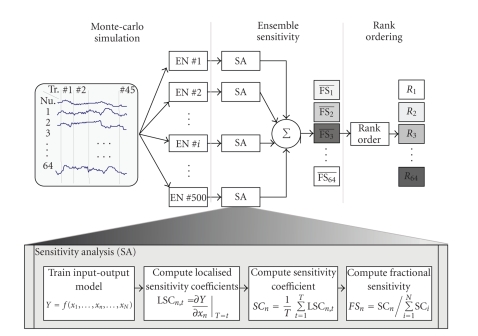
An ensemble of *M* unique nonlinear models was trained using Monte Carlo simulations; each of these models related the input neuronal activity to the output behavior with high accuracy. In order to assess the contribution of each model input, *x*_*n*_, to encoding the final output, *Y*, a sensitivity analysis (SA) was performed. For each of the M models, the localized sensitivity coefficients, LSC_*n*_, were calculated by taking the partial derivative of the output with respect to the input activity. The sensitivity coefficients, SC_*n*_, were then computed by taking the average of the localized sensitivity coefficients across all instances of the testing data. The sensitivity coefficients were normalized and expressed as a fraction of the cumulative sensitivity values across all inputs in order to yield the fractional sensitivity coefficients, FS_*n*_. After this sensitivity analysis, neurons were rank-ordered by the mean fractional sensitivities from the ensemble, $\bar{{\text{FS}}_{n}}.\;$ This rank-ordered list was passed to filter to decode the motor task.

For this study, the BMI experimental paradigm of decoding wrist angle using neural recordings from the primary motor and premotor cortex of a nonhuman primate is examined. While the input-output models used in this study are specific to the specific motor task problem, the methods proposed in this work can be generalized and applied to select neurons for more general decoding task. Figure 1 shows a block diagram that summarizes the steps involved in rank-ordering neurons based on ensemble fractional sensitivities.

## 2. Methods

### 2.1. Experimental Setup

This work uses data collected from experiments carried out at Arizona State University (ASU, Tempe, AZ), which have been the subject of previous studies [20]. The experimental protocols were reviewed and approved by the ASU Institutional Animal Care and User Committee (IACUC). Details of the experimental protocol can be found in [21] and are summarized here briefly.

**Figure 2:**
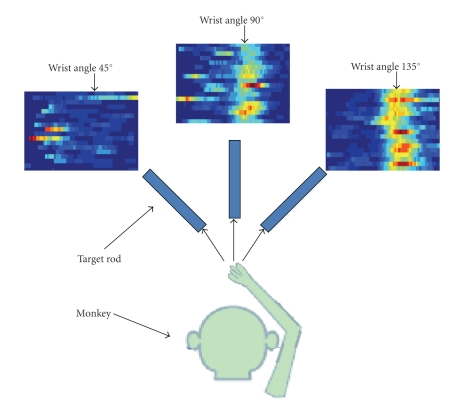
Two rhesus monkeys were trained to reach towards and rotate its wrist to grasp a rectangular target positioned at one of three orientations (45°, 90°, 135°).

Two male rhesus monkeys (Monkey A & B) were trained to reach towards and rotate its wrist to grasp a rectangular target positioned at one of three orientations (45°, 90°, or 135°) in the frontal plane (Figure 2). Visual cues were used to initiate and end the movement. Neural recordings were obtained using a Thomas recording system with 5-channel microelectrode manipulator system. With this recording setup, the penetration depth of each electrode was independently adjusted to capture maximal task-related activity from the primary motor cortex (M1) hand area, dorsal premotor cortex (PMd), and ventral premotor cortex (PMv). With 5 recording sites, approximately 10–15 spike sorted units could be isolated in one session and a total of 297 single units over 63 sessions. Data from each single unit was recorded for 15 trials per movement type and a total of 15×3 = 45 trials for every single unit. In order to demonstrate the advantages of using an ensemble of models for sensitivity analysis, two different analyses were conducted and reported in this manuscript: (1) rank-ordered neurons were used in decoding filter to measure the decoding accuracy; and (2) noisy, task-unrelated single units were identified from a population. Monkey B performed the task poorly and hence its data was only used in the second analysis.

### 2.2. Modeling the Input-Output Relationship

In order to assess the information content of a neuron, that is, how the motor task is encoded, a nonlinear filter was chosen to model the input neuronal activity to the corresponding output kinematic variable [22]. A popular nonlinear filter is the Artificial Neural Networks (ANNs), which are widely accepted as universal approximators [23, 24] and hence used here. ANN consists of an interconnected group of artificial neurons which process information using a connectionist approach to computation.

**Structure of the Neural Network Models**

We designed a multilayer, feed forward ANN with a single hidden layer. The hidden layer was designed with a log sigmoidal transfer function, while the output layer was designed with a linear transfer function. The wrist angle 45, 90, and 135 were normalized to −1, 0, and 1. A linear transfer function at the output layer was chosen because, (1) it was able to fit the input-output data accurately, and (2) it is differentiable, which is a prerequisite for sensitivity analysis. There is no memory in the model. The model topology is described by the following equation:

(1) $$\begin{array}{c} y_{k}=\overset{H}{\underset{j=1}{\sum }}w_{jk}^{2}g\left(\overset{N}{\underset{i=1}{\sum }}w_{ij}^{1}x_{i}+b_{j}^{1}\right)+b_{k}^{2}\\ g\left(x\right)=\frac{1}{\left(1+e^{-x}\right)}, \end{array}$$

where *N* is the number of input neurons, *H* is the number of hidden layer neurons, *k* is the output neuron (there is only one output neuron corresponding to the wrist angle, so *k* = 1), *x*_*i*_ are the neuronal firing rates, and *w*_*i* *j*_^1^, *b*_*j*_^1^, *w*_*j* *k*_^2^, and *b*_*k*_^2^, are the connection weights between input and hidden layer, biases of the hidden, connection weights between the hidden and the output layers, and the biases of the output layer, respectively. The networks were trained using the scaled conjugate gradient (SCG) algorithm with early validation stop to prevent overfitting. The neural networks were trained offline using MATLAB 7.4 (Mathworks Inc.).

Training, validation, and testing data were selected from mutually exclusive trials—with 8 trials used for training, 3 trials used for validation, and 4 trials used for testing. Only those models with high predictive accuracies (>80%) were carried forward for analysis.

### 2.3. Fractional Sensitivity Analysis

Once the ANN model has been trained to map the input (neuronal firing rates) to the output (wrist angle), the contribution of each model input towards the final output is assessed. This is a direct metric of the contribution of the individual inputs as it pertains to decoding of the output variable.

Intuitively, the effect of each neuron on the prediction outcome can be studied by determining the change in outcome due to an infinitesimal change in each input neuron's activity. Mathematically, this is equivalent to taking the partial derivative of the output with respect to the input activity and is referred to as sensitivity analysis [25]. The sensitivity coefficients are a function of the inputs in addition to the connection weights and biases of the trained model. For a given instance of the testing data, the *localized sensitivity coefficients* (LSC_*n*_) were calculated as

(2) $$\begin{array}{cc} {\text{LSC}}_{n\;} & =\left|\frac{\partial y}{{\partial x}_{n}}\right|=\;\left|\frac{\partial }{{\partial x}_{n}}\overset{H}{\underset{j=1}{\sum }}W_{jk}\text{g}\;\left(\overset{N}{\underset{i=1}{\sum }}{\text{w}}_{ij}x_{i}+b_{j}\right)+\;b_{k}\right|\\ & =\left|\overset{H}{\underset{j=1}{\sum }}W_{j}\frac{\partial }{{\partial x}_{t}}\text{g}\left(\overset{N}{\underset{i=1}{\sum }}{\text{w}}_{ij}x_{i}+b_{j}\right)\right|. \end{array}$$

In order to expand the derivative terms we let

(3) $$\begin{array}{cc} g\left(P_{j}\right) & =\frac{1}{1+e^{-P_{j}}}\;\;\;\;\text{where}\;P_{j}=\overset{N}{\underset{i=1}{\sum }}w_{ij}x_{k}+b_{j}\\ ∴\frac{\partial g}{\partial P_{j}} & =\frac{e^{-P_{j}}}{{\left(1+e^{-P_{j}}\right)}^{2}}=\left(1-\frac{1}{1+e^{-P_{j}}}\right)\left(\frac{1}{1+e^{-P_{j}}}\right).\;\\ & =g\left(P_{j}\right)\left[1-g\left(P_{j}\right)\right]. \end{array}$$

Therefore, the localized sensitivity coefficients become,

(4) $${\text{LSC}}_{n}=\left|\overset{H}{\underset{j=1}{\sum }}w_{j}w_{\eta j}g\left(\sum_{i=1}^{N}w_{ij}x_{i}+b_{j}\right)\left[1-g\left(\sum_{i=1}^{N}w_{ij}x_{i}+b_{j}\right)\right]\right|.$$

Note that although the weights and biases were fixed once the model was trained, the value of the sensitivity coefficients (as described by (5)) was dependent on the actual data point and thus varied for different trials on which the model was tested. Different input vectors are represented by the time index *t* in (6).

The *sensitivity coefficient*s (SC_*n*_) for each neuron were calculated by taking the average value across all instances of the training data.

(5) $${\text{SC}}_{n}^{}=\frac{1}{T}\overset{T}{\underset{t=1}{\sum }}{\text{LSC}}_{n,t}^{},$$

where *T* is the size of the training set.

The sensitivity of each input was expressed as a fraction of the cumulative sensitivity values across all the inputs. This *fractional sensitivity* (FS_*n*_) was calculated as,

(6) $${\text{FS}}_{n}^{}=\frac{{\text{SC}}_{n}^{}}{\sum_{i=1}^{N}{\text{SC}}_{i}},$$

where *N* is the total number of neurons and *n* is the neuron whose fractional sensitivity is being computed.

### 2.4. Ensemble Selection of Neurons

To select a neuronal population for decoding the motor task, we rank-order neurons by their fractional sensitivity and then select as many neurons from the rank-ordered list as needed to achieve the desired accuracy levels. 

A major drawback of using such model-based approaches lies in the fact that the sensitivity values are dependent on the training data. Even with models that achieve high levels of predictive value, the sensitivity analysis will result in unique solutions that may not correspond to a solution that can be generalized to new datasets. This is because any single solution may only find a local minima and not a globally optimal solution for the entire dataset. Therefore, to avoid getting stuck in local minima, the fractional sensitivities were computed over an *ensemble* of *M* independently trained models. Each model was trained with a different training data set which is a randomly drawn subset of trials from a pool of total training dataset. 

We propose, and later justify, that the *mean* of the distribution of fractional sensitivities for a neuron computed across different models is a good global estimate of its information content. Additionally, we place confidence intervals on this estimate. Neurons are then selected based on ranks of these *mean fractional sensitivities *( $\bar{{\text{FS}}_{n}}$ ).

**How to determine the number of models used in the ensemble?**

**Figure 3:**
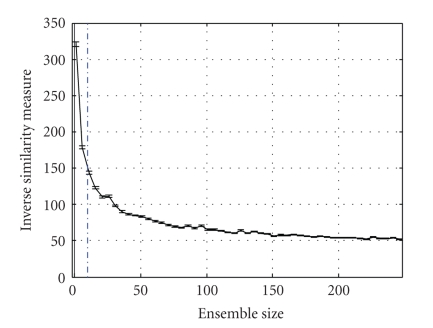
The weighted average of absolute differences (inverse similarity measure) between a master rank-ordered list (computed for *M* = 500) and rank-ordered lists for varying ensemble sizes was used to choose an optimal ensemble size of *M* = 10.

A master rank-ordered list was generated using an ensemble of 500 unique models. Assuming that the spike trains from one single unit is independent from each other, we performed Monte-Carlo simulation across neurons to generate data to train multiple. Rank-ordered lists for varying ensemble sizes were then computed, and a weighted average of absolute differences with the master rank-ordered list was used as a similarity measure. Neurons with higher ranks were given more weight. From Figure 3, we choose an ensemble size (*M*) of 10 for this particular task, as a trade-off between good convergence of the similarity of the rank-ordered list to the master list and the computational burden of too large an ensemble size.

### 2.5. Simulation of Noisy Environment

In order to provide evidence that the success rate when noisy neurons are identified using sensitivity values from an ensemble models is higher than single model, a priori knowledge on which neurons are noisy is necessary. We simulated spontaneously uncorrelated single units and added these noisy neurons to the input space. Spontaneously firing units were generated using a Poisson random process, with the rate constant (*λ*) estimated from the average neural activity during the “no stimulus” period.

For this simulation study, we systematically varied the percentage yield of task-related units recorded from the array by adding a fixed number of noisy uncorrelated neurons. This analysis was carried out on datasets from both the Monkeys (A & B) and the results were reported.

### 2.6. Decoding Filters

Using the rank-ordered lists of neurons from the ensemble of models, decoding of the motor task was performed using a nonlinear ANN-based filter. For all practical purposes, the number of neurons in the feature space is limited by the number of recording sites on an implanted microelectrode array. Therefore, in order to more closely approximate this scenario, which mimics a multielectrode array that simultaneously captures and records from multiple neurons, the decoding filters were trained using a randomly sampled subset of neurons (*N* = 64) from the entire neuronal population of 297 neurons. This analysis was only performed on the data from Monkey A. Monkey B performed the task poorly and hence its data was not used in this specific analysis.

### 2.7. Statistics

Nonparametric tests were performed to test for differences in the median decoding accuracy using a single model for neuron selection versus an ensemble of models. The null hypothesis tested was that the median decoding accuracy obtained using an ensemble of models was equal to the median decoding accuracy from each of the individual models.

## 3. Results

### 3.1. Modeling Accuracy

Before performing sensitivity analysis to assess the contribution of a neuron, it is first necessary to show that models used to relate the input neuronal activity to the output behavior are accurate.

Table 1 shows the confusion matrix for the decoding accuracies for 10 models (mean accuracy = 88.52%, SE = 4.5%). Only those models with high predictive accuracies (>80%) were retained as part of the ensemble.

### 3.2. Distribution of Fractional Sensitivities

**Figure 4:**
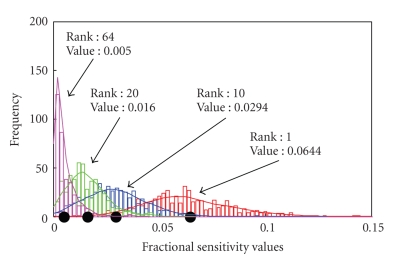
Distribution of fractional sensitivities for four exemplary neurons, using an ensemble of 500 nonlinear models. Note that although an ensemble of 10 models is used for neuron selection, 500 models are shown here to illustrate that fractional sensitivities cluster around a mean value. Histogram plots arranged according to the rank of the neurons (marked on top right in each plot). Mean fractional sensitivity for each neuron is represented by black scatter points.

Figure 4 shows the distribution of fractional sensitivities for four exemplary neurons as returned by each of the individually trained models. Fractional sensitivity values from 500 models were used to generate smooth distribution function (Figure 4). The fractional sensitivities appear to cluster around a common mean value, which leads us to use it as the representative fractional sensitivity value for the entire ensemble.

### 3.3. Ranking of Neurons Using Fractional Sensitivities

**Figure 5:**
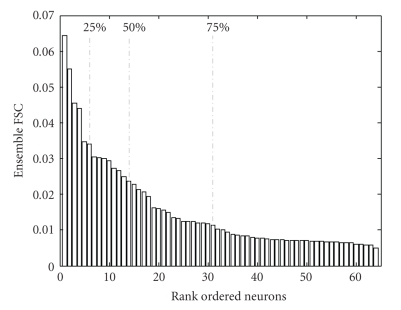
Distribution functions of the contributions of each of the 64 neurons to the total sensitivity, as ranked by their mean fractional sensitivities. The graph is positively skewed and the values drop exponentially. The distribution functions for each model are similar, with the top 5, 13, and 30 neurons containing 25%, 50%, and 75% of the total information content, respectively.

Figure 5 shows the contribution of each of the 64 neurons in a subpopulation to the total sensitivity presented in a ranked order. Mean fractional sensitivities are used to rank the neurons. Top 5 neurons contribute 25%, the top 13 neurons contribute 50%, and the top 30 neurons contribute 75% of the total sensitivity.

### 3.4. Merit of Ensemble Models over a Single Model

**Figure 6:**
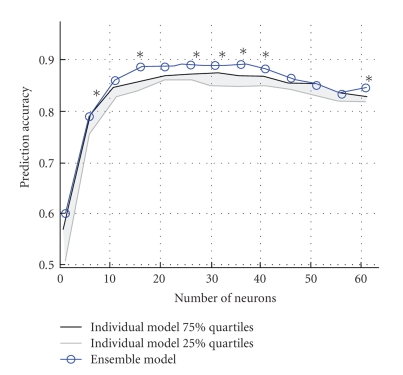
The upper quartile and lower quartile prediction accuracies across 10 individual rank models are plotted as a function of the number of neurons. Using an ensemble of 10 trained models resulted in statistically significant higher (*,  *P* < .05) decoding accuracies than if using a single model alone. Asterisks above the plots indicate that the data points for which the decoding accuracies are statistically different. This analysis was done on data obtained from Monkey A.

In order to contrast between the neuron ordering from ensemble versus single model, decoding accuracies were computed in following two scenarios, (a) when the neurons were rank-ordered using sensitivity values computed using an ensemble of 10 models (Figure 6: blue curve), and (b) when the neurons were rank-ordered using sensitivity values from a single model. This method was repeated for all the 10 models separately which resulted in 10 individual decoding accuracy curves. The 25% and 75% quartiles are shown in Figure 6 (with grey and black curves). 

Friedman's 2-way ANOVA was performed to test the hypothesis that the median decoding accuracy obtained using an ensemble of models was equal to the median decoding accuracy from each of the individual models. A small, albeit statistically significant (*P* < .05) increase of around 5% is obtained in the decoding accuracies when an ensemble approach was used. 

Additionally, Kruskal-Wallis tests were conducted (*P* < .05) to identify the number of input neurons for which decoding accuracies are statistically different for ensemble models versus single models. Asterisk above the plots in Figure 6 indicate that the data points for which the decoding accuracies are statistically different.

### 3.5. Merit of Sensitivity-Based Ranking over Other Ranking Methods

**Figure 7:**
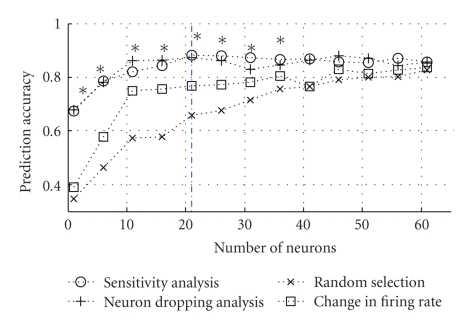
Ranking neurons based on the ensemble fractional sensitivities resulted in decoding accuracies 10%–20% greater than when randomly selecting neurons or ranking-based on firing rates alone. The vertical blue line marks the optimal number of neurons, beyond which prediction performance decreases for the sensitivity ranking-based approach.

Figure 7 compares the different approaches used to rank order the neurons, (1) random selection of neurons, (2) rank-ordered using change in firing rates, (3) rank-ordered using a neuron dropping approach suggested in Figure 7, and (4) rank-ordered using ensemble fractional sensitivities. 

Ranking neurons based on the ensemble fractional sensitivities resulted in decoding accuracies 10%–20% greater than when randomly selecting neurons or ranking-based on firing rates alone. The decoding accuracies were also comparable to those obtained when neurons were ranked using a neuron dropping analysis [7]. From Figure 7, the average decoding accuracy was as high as 90% if the neurons were ranked using ensemble fractional sensitivities, with a peak accuracy for *N* = 21. However, average decoding accuracy only went up to 80% if randomly selecting the same number of neurons or ranking-based on firing rates. All approaches converge for a higher number of neurons.

### 3.6. Simulation Study: Robustness of Ensemble Approach in a Noisy Environment

In order to assess the robustness of the ensemble approach compared to a single model, we measured how accurately each method was able to correctly classify the original task-related units in the presence of varying amounts of noisy neurons. For example, a 10% yield of task-related units implies that in a neural population of 64 units, 7 are task-related and the rest are simulated noise.

Tables 2(a) and 2(b) compare the accuracy for both neuron selection approaches for different array yields, in Monkey A and B, respectively. The ensemble approach consistently recovered the task-related neurons with higher accuracy (around 25% on an average) than the single model approach. The accuracy was defined as percentage of original input neurons which were correctly identified as task-related by the neuron selection algorithm. Mean and standard error over 10 repetitions (each repetition starts with different original subset of neurons) are reported in Tables 2(a) and 2(b). 

## 4. Discussion and Conclusion

In this work, we established a novel approach to rank-order a population of neurons by their relative contribution to the decoding of a motor task, dubbed “*fractional sensitivity”.* Although sensitivity analysis has been previously used to ascertain the relative importance of each input variable (in this case neurons), one common problem associated with previous model-based sensitivity approaches is the inability to generalize its rank list over a new dataset. This is especially true when there is redundancy in the input space in terms of encoding of the final output variable, as is the case with most complex motor tasks. 

### 4.1. Benefits of Using an Ensemble of Trained Models

Our results show that using an ensemble of trained models helps to offset this problem of generalization. The benefits of using an ensemble model are quantified by an increase in overall decoding accuracy. As seen in Figure 6, there is an increase of up to 5% in the decoding accuracy when an ensemble of models is used as compared to a single model. Although the benefits of an ensemble approach are debatable on the grounds of such a modest increase in decoding accuracy, there are systems wherein identification of noisy inputs in itself may be more important than the decoding accuracy. In such scenarios, the percentage of noisy neurons that are correctly identified can be used as a quantifiable measure of the benefits of using an ensemble approach. The results of our simulation study (Tables 2(a) and 2(b)) confirm that an ensemble approach to identification of noisy neurons is 25% more accurate than when using a single model. 

We also hypothesize that the particular motor task discussed in this paper (i.e., wrist rotation) is relatively less complex, and hence the benefits of using an ensemble of trained models are not as evident. Further investigations are needed involving more complex, nonlinearly encoded motor tasks, such as dexterous finger manipulations, to support or reject this hypothesis.

### 4.2. Model Dependent versus Model Independent Approaches

Model independent approaches do not make any assumption about the classification model used during the selection process. Methods such as Mutual Information estimate the “uncertainty reduction” in one variable when another variable is provided. However, this requires estimation of joint and marginal distributions, which is computationally and time intensive. In BMI applications where the neural properties change continuously, models must be retrained repeatedly and this increases the computational overhead. Model dependent approaches, on the other hand, while computationally robust, lack generalization since the input features that get selected may not represent the entire training data, especially if there is data redundancy. 

Our approach of using an ensemble sensitivity analysis tackles both issues by offering a computationally robust model dependent approach that also results in generalized feature selection. Although in this paper an Artificial Neural Network model accurately describes the input-output map between neurons and wrist angle kinematics, this approach is not limited to a specific decoding model and can generalize to other filter choices as well. This will be largely dependent on the scope of the problem and complexity of the input feature space.

### 4.3. Neuron Dropping versus Sensitivity Analysis

In terms of assessing the merits of a sensitivity analysis-based approach to ranking neurons, Figure 6 shows that the improvement in prediction accuracy when neurons are selected using Sensitivity Analysis is much greater than when randomly selecting neurons, but comparable to that when using a neuron dropping analysis [7]. 

It is important to note, however, that although there is a negligible difference in decoding accuracy, the neuron dropping approach employs a computationally burdensome, greedy algorithm. Relevant neurons are identified by systematically dropping them from the input space and measuring the corresponding change in decoding accuracy, with a rise in accuracy after a neuron is dropped as the stop criterion. In comparison, our proposed ensemble sensitivity analysis approach returns a rank-ordered list of neurons by looking at the entire input space at once and thus uses a fewer number of iterations. This computational saving is important in resource-limited BMI applications.

### 4.4. Neuron Selection for Closed Loop BMI

It should be noted that the results of this paper were restricted to offline decoding of the motor the task based on *a priori* knowledge of the time of movement. In a chronic implanted BMI, properties of the input space change dynamically because of movements of electrode arrays and noise induced by degradation at the electrode-tissue interface. This necessitates retraining of the algorithms for each session, and since the neuron selection approach proposed in this paper is an integral part of the training process, it can conceivably be used to select the best neurons every time after the training is complete.

Furthermore, recent closed loop BMIs developed by Carmena et al. [2], Schwartz et al. [6, 26], and Serruya et al. [27] have found that, owing to the “plasticity of brain”, neurons can modulate their firing properties over time and eventually result in higher decoding accuracies overall. Although this phenomenon is not completely understood, the method of neuron selection proposed in this paper extracts the contribution of each input after an input-output map has already been obtained. Therefore, any change in the firing rate of the neurons, and its effects on the prediction of the motor task, will be automatically addressed. As a result, the results of this work generalize well to future closed-loop BMI experiments.

## Figures and Tables

**Table 1:** Confusion Matrix of Decoding Accuracies.

Wrist angle	45 degrees	90 degrees	135 degrees
45 degrees	**92.9%**	7.1.%	0.0%
90 degrees	1.6%	**92.1%**	6.3%
135 degrees	0.0%	19.3%	**80.7%**

Columns and rows represent the actual and predicted class, respectively.

**Table:** (a) Comparison of an ensemble of models versus single model in a noisy environment (Monkey A).

% Yield of task-related units (%)	Single model (%)	Ensemble of models (%)
10	51.7 ± 4.6	83.3 ± 0.0
20	58.3 ± 6.9	91.7 ± 0.0
30	67.9 ± 2.4	93.7 ± 0.7
40	66.0 ± 3.5	94.8 ± 0.8
50	69.7 ± 1.6	94.4 ± 1.0
60	75.3 ± 1.3	94.3 ± 1.3

**Table:** (b) Comparison of an ensemble of models versus single model in a noisy environment (Monkey B).

% Yield of task-related units (%)	Single model (%)	Ensemble of models (%)
10	53.3 ± 6.5	83.3 ± 0.0
20	58.3 ± 3.5	90.8 ± 0.8
30	65.8 ± 4.3	92.6 ± 0.9
40	68.0 ± 4.9	93.2 ± 1.2
50	66.3 ± 3.7	96.6 ± 0.3
60	73.2 ± 2.0	96.6 ± 0.4
